# Neuroligin Plays a Role in Ethanol-Induced Disruption of Memory and Corresponding Modulation of Glutamate Receptor Expression

**DOI:** 10.3389/fnbeh.2022.908630

**Published:** 2022-05-26

**Authors:** Jacqueline K. Rose, Michael Butterfield, Joseph Liang, Mahraz Parvand, Conny H. S. Lin, Catharine H. Rankin

**Affiliations:** Brain Research Centre, University of British Columbia, Vancouver, BC, Canada

**Keywords:** ethanol, *C. elegans*, glutamate receptor, neuroligin, memory blackout

## Abstract

Exposure to alcohol causes deficits in long-term memory formation across species. Using a long-term habituation memory assay in *Caenorhabditis elegans*, the effects of ethanol on long-term memory (> 24 h) for habituation were investigated. An impairment in long-term memory was observed when animals were trained in the presence of ethanol. Cues of internal state or training context during testing did not restore memory. Ethanol exposure during training also interfered with the downregulation of AMPA/KA-type glutamate receptor subunit (GLR-1) punctal expression previously associated with long-term memory for habituation in *C. elegans*. Interestingly, ethanol exposure alone had the opposite effect, increasing GLR-1::GFP punctal expression. Worms with a mutation in the *C. elegans* ortholog of vertebrate neuroligins (*nlg-1*) were resistant to the effects of ethanol on memory, as they displayed both GLR-1::GFP downregulation and long-term memory for habituation after training in the presence of ethanol. These findings provide insights into the molecular mechanisms through which alcohol consumption impacts memory.

## Introduction

The consequences of alcohol consumption in humans include deficits in decision-making, problem-solving, and in learning and memory (Leckliter and Matarazzo, 1989; Selby and Azrin, 1998). Alcohol intoxicated individuals show impaired performance on tasks such as learning word lists (Grant, 1987), short- and long-term logical memory (Selby and Azrin, 1998), and general working memory (Ambrose et al., 2001). Further, people who have experienced alcohol-induced blackouts continue to show impaired recall the next day when sober (Jackson et al., 2021). At the neuron level, cellular correlates of memory (primarily long-term potentiation and depression) are both attenuated by ethanol exposure (White et al., 2000; Chandler, 2003; Izumi et al., 2005; Avchalumov and Mandyam, 2020; Mira et al., 2020). Further, chronic alcohol exposure reportedly causes significant changes in expression of the overall brain transcriptome in prefrontal cortex neurons of mice (Liu et al., 2022). Although many studies have identified neural effects of alcohol consumption, the mechanism(s) by which alcohol affects learning and memory still requires investigation.

Ethanol has been found to interact with several neurotransmitter systems. The role of GABA in mediating the intoxication effect of ethanol are well-established (see Kumar et al., 2009; Chandler et al., 2017). Glutamate has also been shown to be affected by ethanol (see Chastain, 2006; Rao et al., 2015). A number of correlations have been reported between ethanol-induced changes in glutamate receptor activity and the behavioral effects of ethanol (i.e., Harris et al., 1998; Chandler, 2003; see Valenzuela, 1997, or Woodward Hopf and Mangieri, 2018). Gioia and McCool (2017) reported an inhibitory effect of ethanol on neurons of the basolateral amygdala in mice, an area involved in fear conditioning. Gioia et al. (2017) found that ethanol had a negative effect on vesicle recycling proteins at glutamatergic synapses in part due to an α-amino-3-hydroxy-5-methyl-4-isooxazole (AMPA) receptor-mediated form of post-synaptic facilitation. As well, Salling et al. (2016) showed that animals that consumed ethanol showed increased AMPA receptor expression in the central amygdala suggesting some of the effects of ethanol on learning and memory may be mediated by modulation of glutamate signaling.

Ethanol affects behaviors across a variety of species and may do so through orthologous gene pathways (Crabbe et al., 1994). Given the effects of ethanol on memory and on glutamate signaling reported in mammalian brains, the current study investigated the effects of ethanol on long-term habituation, a glutamate-dependent form of long-term memory in the *C. elegans* model system (Rose et al., 2002, 2003). Habituation is a non-associative form of learning that is observed as a decrease in response to repeated stimulation. Long-term memory (>24 h) for habituation is seen following a spaced training protocol (Rose and Rankin, 2001). This long-term memory for habituation is glutamate-dependent and is correlated with decreased punctal expression of GLR-1, an AMPA/KA-type glutamate receptor subunit (Rose et al., 2003). The current study examined whether ethanol exposure during training would impair long-term memory and block the decrease in GLR-1 punctal expression levels after training. A surprising discovery that GLR-1 punctal expression is increased in untrained animals exposed to ethanol led to investigation of a possible role of the postsynaptic cell adhesion protein neuroligin (NLG-1). Results indicate that ethanol exposure during training disrupts the formation of long-term memory, and that NLG-1-mediated regulation of GLR-1 levels may underlie the memory deficits observed.

## Methods

### Animals

Worms were maintained on nematode growth medium (NGM) agar plates seeded with *Escherichia coli* (OP50) and maintained at 20°C. *C. elegans* wild-type N2 Bristol and *nlg-1*(ok259) strains were obtained from the Caenorhabditis Genetics Center (University of Minnesota, Minneapolis, MN). KP1580 *pglr-1*::GLR-1::GFP was provided by J. Kaplan (Harvard University, Boston, MA). RM3389 *nlg-1*(ok259); p*nlg-1*::NLG-1::YFP was provided by J. Rand (Oklahoma Medical Research Foundation, OK).

### Ethanol Plate Preparation

To ensure consistent agar conditions across days, single plates were weighed on training day. The appropriate amount of ethanol (100%) was added to the agar for each plate to the desired concentration (0.2 M, 0.4 M, or 0.6 M), then plates were wrapped in parafilm for 1–2 h to allow equilibration of ethanol into the agar. Previous work using gas chromatography found that 0.5 M ethanol exposure in *C. elegans* corresponds to a clinically relevant concentration of approximately 0.3–0.4% blood alcohol concentration (Alaimo et al., 2012). Ethanol concentrations at this level remain low enough to effectively measure behavior without producing immobility (Davies et al., 2003; Alaimo et al., 2012). Testing plates were seeded with E. coli 1 day prior to testing.

### Behavior Testing and Analysis

Behavioral observations of small groups of worms were made using a Wild Leitz stereomicroscope (Zeiss, Canada). Petri plates containing 15–20 worms received a tap (~1.5 N force) delivered to the side of the Petri plate via a copper rod connected to a 6-V electromagnetic relay. This “tapper” was activated with a Grass S88 (Quincy, MA) stimulator set to deliver a single tap stimulus. Worm locomotor responses were video recorded for analysis. The reversal response magnitude (i.e., the distance the worm crawled backwards in response to the tap stimulus) was scored using stop-frame video analysis. Response tracings were quantified in NIH Image (version 1.63). Two-way mixed ANOVAs with Fisher's protected least significant difference (PLSD) *post-hoc* tests were used to test for statistical significance. If only two groups were analyzed, then student *t*-tests were employed.

### Long-Term Habituation Training and Testing

Four-day-old worms (~90–96 h after eggs were laid at 20°C) were transferred to either ethanol-containing plates or ethanol-free training plates (~20 worms/plate) and allowed 1 h to recover from transfer and to absorb ethanol. To deliver uniform mechanosensory stimuli to a large number of training plates simultaneously, training plates were placed in a Tupperware container and dropped from a height of ~5 cm onto a tabletop. Training consisted of four blocks (20 drops given at a 60 s interstimulus interval, ISI) with training blocks separated by 1-h rest periods. Untrained groups were dropped once immediately at the end of the trained groups to control for any potential influence of pseudoconditioning due to responding to novelty. One hour after training, all worms were transferred to ethanol-free plates containing a small amount of OP50 *E. coli* (50–100 μL). For conditions where worms were exposed to ethanol for seven hours after training, both trained and untrained worms were transferred to fresh ethanol plates 1 h after training and to ethanol-free plates at the end of the ethanol exposure time.

To test retention, 24 h after habituation training 15–20 worms (5-days-old from egg-laying) were transferred to either a plain agar plate (normal long-term retention) or a plate containing ethanol (0.4 M to test for state conditioning and 0.05 M for context conditioning) and given 1 h to acclimate. After a 6-min pretest period, worms were given five tap stimuli at a 60 s ISI and the responses were recorded. Prior to each tap stimulus, plates were repositioned on the microscope stage to maximize the number of worms in the field of view meaning that responses were captured as a population measure, randomized across worms.

### Testing Short-Term Habituation

Four-day-old worms were transferred to a test plate containing 0.0 M, 0.2 M or 0.4 M ethanol. A tap stimulus was delivered to the side of the plate either at a 10 s ISI or a 60 s ISI and worm locomotor responses were video recorded and analyzed (see Behavior Testing and Analysis).

### Confocal Fluorescence Imaging

To quantify levels of GLR-1::GFP punctal expression, worms were paralyzed in 12 μL of 50 mM sodium azide solution on a sterile glass microscope slide. Worms were then placed on a 2% agar pad and covered with a 1.5 thickness glass coverslip for imaging. Images of GLR-1::GFP clusters along the ventral nerve cord were collected with an Olympus FV1000 confocal microscope (Leica SP8 for the experiments examining effects of ethanol only on GLR-1 expression and Supplementary Figure 1) with optical sections collected at 0.5 μm intervals using a 63 × oil immersion lens and consistent confocal microscope settings: gain = ~1.0; PMT = 600 (+/−50), laser = ~1.0%. FIJI and ImageJ v1.33 were used to measure area of fluorescence punctal expression from Z-projected image stacks.

### RNA Isolation and Quantification

*C. elegans* RNA was isolated using Trizol extraction (two biological replicates at three dilutions performed in triplicate for each group). RNA was reverse transcribed with SuperScript III First-Strand Synthesis System for RT-PCR with oligo(dT) primers according to the manufacturer's protocol (Invitrogen, Carlsbad, CA). The ΔΔCT method of quantification was performed on an ABI 7000 (Applied Biosystems, Foster City, CA) using SYBR GREEN. For *glr-1* and *act-1* (reference gene) the following primers were employed: forward GGA GAG GTT CTG GTT TTGATT GA and reverse TCG AGT ACG AAG ATG TCT CCA AAG for *glr-1*; forward TTG CCC CAT CAACCA TGA A and reverse CTC CGA TCC AGA CGG AGT ACT T for *act-1* (Ebrahimi and Rankin, 2007).

## Results

### Ethanol Exposure During Training Blocks Long-Term Memory

**Figure 1:**
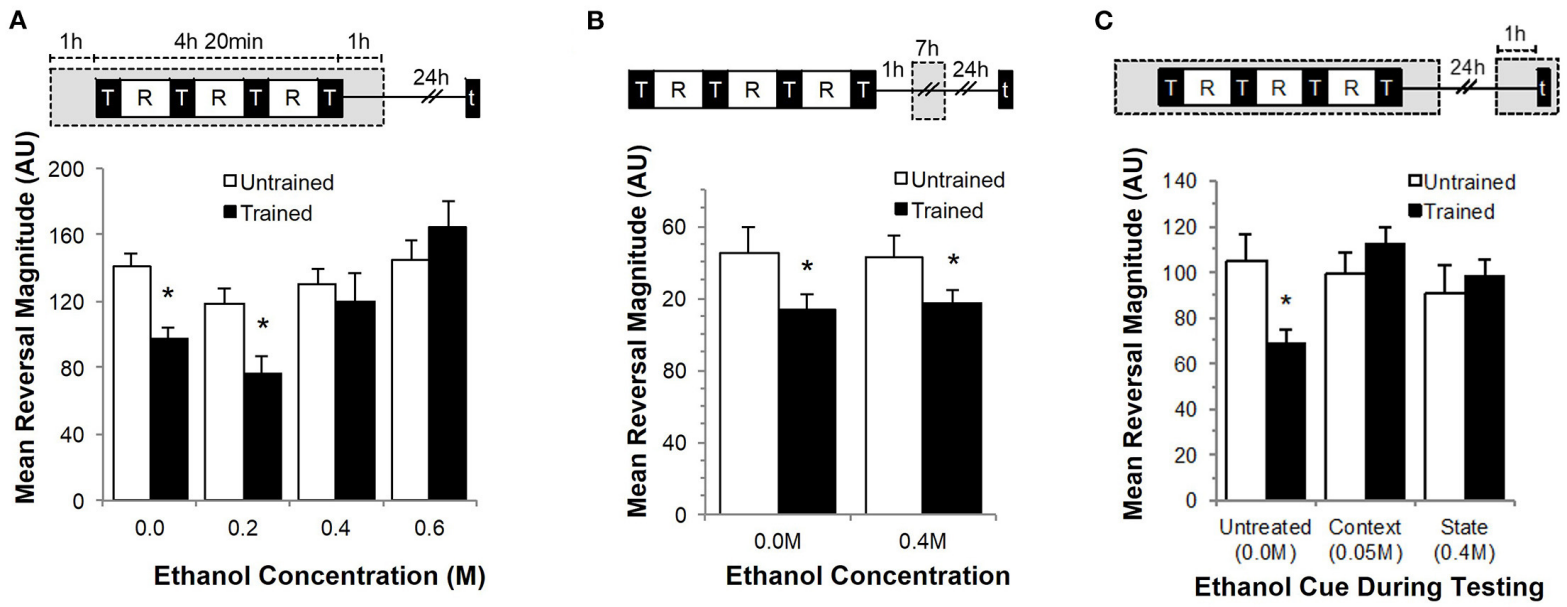
Effects of ethanol exposure during training on long-term memory for habituation. Each graph includes an illustration of corresponding training and ethanol exposure protocols where gray boxes indicate time of ethanol exposure while black boxes indicate training periods denoted by “T” and test periods denoted by “t.” White squares indicate rest periods denoted by “R.” **(A)** Mean reversal response magnitude (± SEM) measured at testing 24 h after exposure to 0.0, 0.2, 0.4 or 0.6 M ethanol exposure compared between trained (black bars) and untrained groups (white bars). **(B)** Mean reversal response magnitude (± SEM) compared between trained (black bars) and untrained groups (white bars) with 7-h ethanol exposure delivered 1-h after training completed. **(C)** Mean reversal response magnitude (± SEM) measured after 0.4 M ethanol exposure during training and re-exposed to either 0.05 M (context cue) or 0.4 M (internal state cue) at testing compared to an unexposed conditioned group. * = *p* < 0.05.

To investigate whether long-term memory for habituation could be disrupted by ethanol in a dose-dependent manner, wild-type worms were trained on agar plates infused with 0.2 M, 0.4 M or 0.6 M ethanol (as previously described in Davies et al., 2003) and tested for memory 24 h later (Figure 1A). Overall, there was a significant interaction between training and ethanol exposure (F_7, 332_ = 2.603, *p* < 0.05). Wild-type worms not exposed to ethanol showed long-term memory as evidenced by significantly smaller reversal responses to the five test taps delivered 24 h after training. Although naïve worms exposed to 0.2 M or 0.4 M ethanol showed a small decrease in reversal magnitude compared to naïve worms not exposed to ethanol, only the worms trained on 0.2 M ethanol showed significant long-term memory 24 h after training (*p* < 0.05). Worms that were trained at higher ethanol concentrations (0.4 M and 0.6 M) showed no evidence of memory for the training (*p* > 0.10). This memory deficit at higher ethanol concentrations was not the result of differences in food quality (ethanol can act as a bactericide for the *C. elegans* OP50 bacterial food source at higher concentrations) as worms tested either “on” or “off” food still showed a significant effect of training (*F*_3, 154_ = 8.840, *p* < 0.001; Supplementary Figure 2).

To test whether the effects of alcohol on memory are specific to exposure during training rather than a generalized memory impairment, worms were first trained in the absence of ethanol, then 1 h after training were exposed to 0.4 M ethanol for a 7-h period (corresponding to the duration of ethanol exposure in the previous experiment). Worms were then tested 24 h after the end of the ethanol exposure (Figure 1B). In this condition, worms exposed to ethanol after training showed a decrement in response to the test taps compared to untrained groups (F_3, 204_ = 3.201, *p* < 0.05; Figure 1B) suggesting that alcohol-induced memory impairment is specific to ethanol exposure during training.

Worms were tested in two cued conditions (context and state conditioning) to determine whether the memory-impairing effect of alcohol during training is the result of a retrieval deficit. Previous research from our lab showed that context conditioning enhances retention of habituation when worms are trained and tested in the presence of the same chemosensory cue (Rankin, 2000; Lau et al., 2013). We tested whether a contextual cue could restore memory for training delivered in the presence of ethanol by testing in the presence of a subtle ethanol cue (0.05 M ethanol; a dose strong enough for the worms to detect the ethanol, but not strong enough to induce an intoxicated state; Bettinger and McIntire, 2004). Alternatively, memory for sensory adaptation training in *C. elegans* that occurred in the presence of ethanol could also be state-dependent (Bettinger and McIntire, 2004). In a *Drosophila* chronic alcohol exposure paradigm, flies only remembered to move away from an attractive odor previously paired with heat shock if they were given a high dose of alcohol again (Robinson et al., 2012). To test whether an inebriated state would cue recall and restore memory, worms were re-exposed to the same ethanol concentration (0.4 M) delivered at training during testing. Memory was only observed in the untreated group in worms that were not exposed to ethanol during testing (F_5, 378_ = 2.813, *p* < 0.05; Figure 1C). Thus, neither the contextual cue nor state-dependent recall was sufficient to restore memory for training that occurred in the presence of ethanol, suggesting that the memory deficit seen with ethanol exposure during training was due to impairments on encoding.

### Short-Term Habituation Affected by Alcohol When Stimuli Presented at Long Interstimulus Intervals

**Figure 2:**
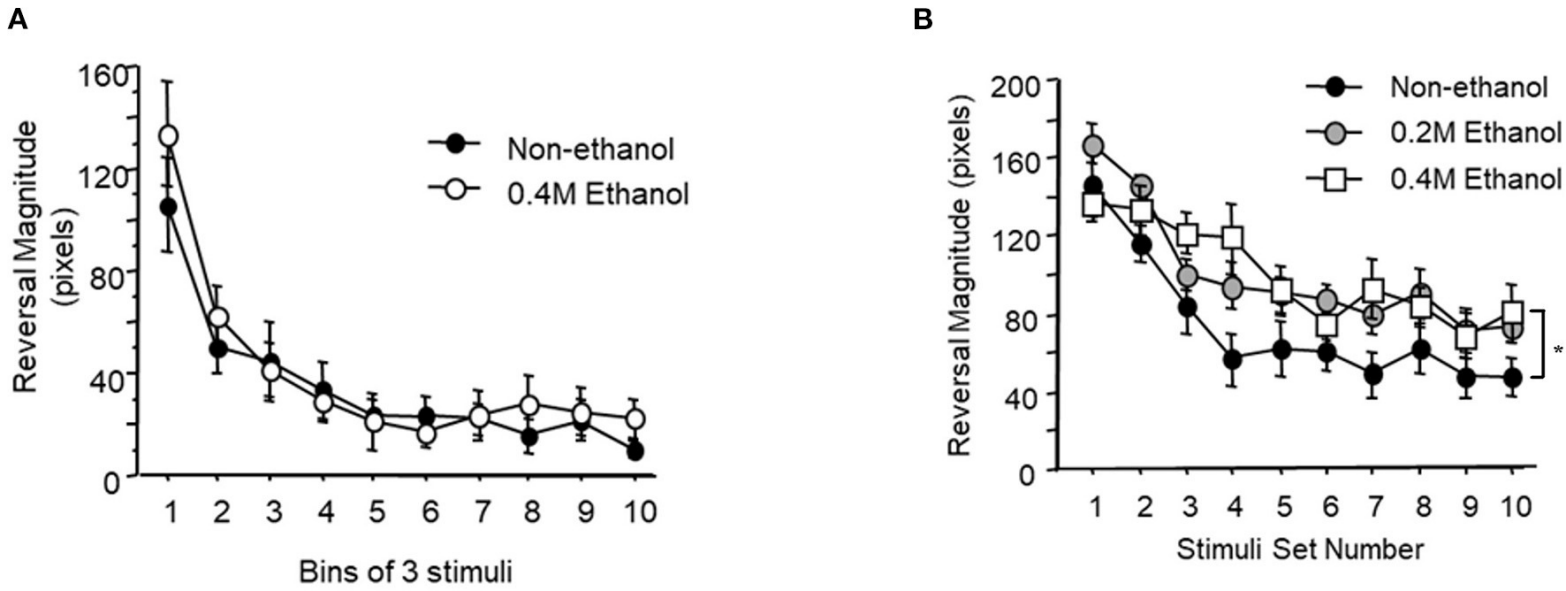
Short-term habituation during ethanol exposure. **(A)** Mean reversal magnitude (±SEM) to 30 tap stimuli delivered at a 10 s interstimulus interval and averaged in bins of three stimuli for control (black circle) vs. worms exposed to 0.4 M ethanol (white circle). **(B)** Mean reversal magnitude (±SEM) to 30 tap stimuli delivered at a 60 s ISI averaged in bins of three stimuli for control (black circle), 0.2 M ethanol exposed worms (gray circle) and 0.4 M ethanol exposed worms (white square).

It is possible that the effects of alcohol on memory for habituation were due to a short-term memory and/or habituation learning impairment. To assess this, worms were given 30 tap stimuli at either a 10 s or a 60 s ISI and the reversal response magnitude was measured for each stimulus presentation to test whether short-term habituation was affected by alcohol. When stimuli were delivered at a 10 s ISI, exposure to 0.4 M ethanol resulted in no significant difference between the untreated control group and the ethanol-treated group in the rate of habituation or the final asymptotic habituated response level (Figure 2A; F_9, 36_ = 0.537, *p* > 0.10).

However, when stimuli were delivered at a longer 60 s ISI, there were significant decreases in habituation between the untreated group and both the 0.2 M and the 0.4 M ethanol treated groups (*p* < 0.05 and *p* < 0.01, respectively). At this longer 60 s ISI the two-way ANOVA revealed a small but significant effect of ethanol exposure (F_18, 378_ = 1.947, *p* < 0.05; Figure 2B). Interestingly, there was only a significant difference between the untreated and 0.4 M ethanol treated groups for asymptotic level of response magnitude (*p* < 0.05), and not between the untreated control and the 0.2 M ethanol treated group. Taken together this data indicates that alcohol exposure does not affect short-term habituation at 10 ISI, but decreases habituation levels at a longer 60 s ISI in both the 0.2 M and 0.4 M ethanol conditions. Despite decreasing 60 s ISI tap habituation levels at 0.2 M, the previous long-term memory experiment (Figure 1A) showed that animals trained in 0.2 M ethanol still exhibited long-term memory. This suggests that the mild impairment effects of alcohol exposure on short-term 60 s ISI habituation does not impede the formation of long-term memory.

### Ethanol During Training Interferes With Decreased Glutamate Receptor Expression Seen With Long-Term Memory for Habituation

**Figure 3:**
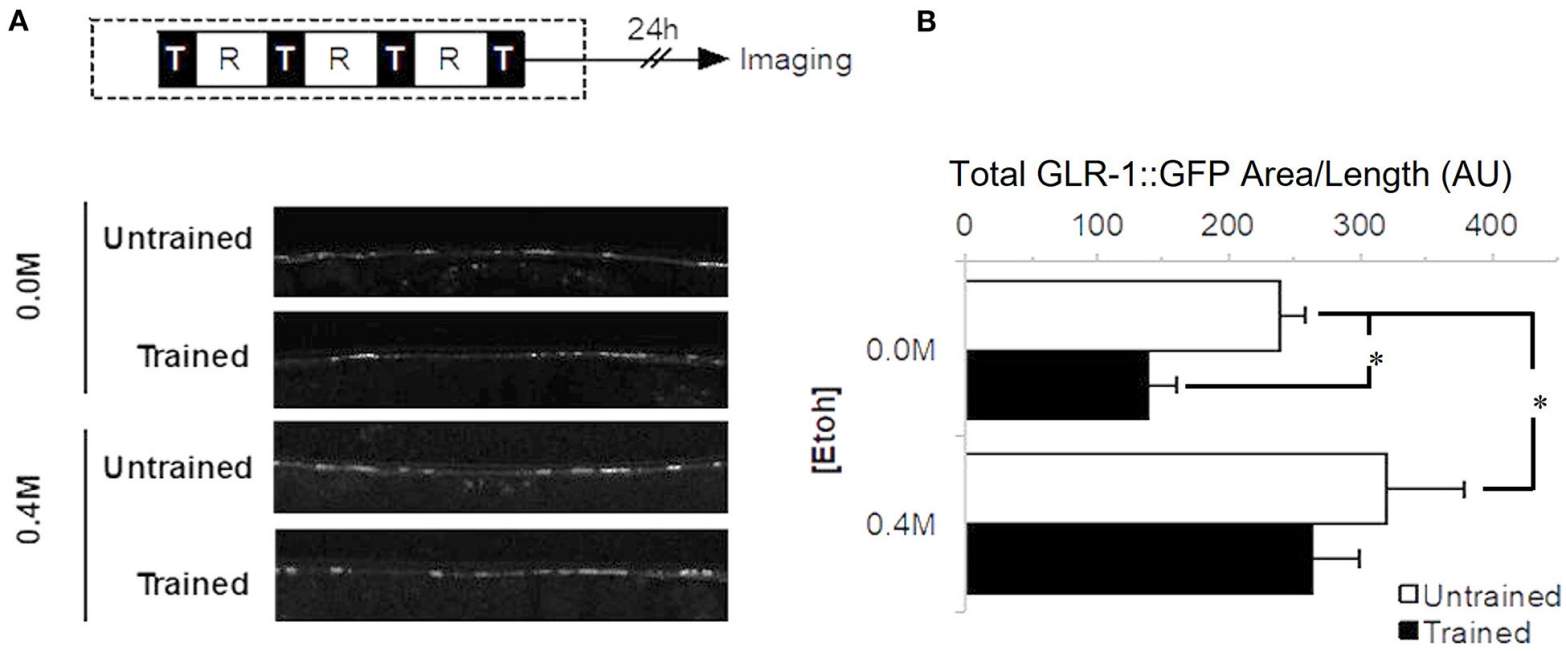
Expression of GLR-1 receptors 24 h after long-term memory training. **(A)** Behavioral protocol whereby long-term memory training is delivered in four blocks (black squares with “T”) separated by 1-h rest periods (white square with “R”). Gray shaded area corresponded to timing of ethanol exposure. Examples of confocal images of GLR-1::GFP expression captured from worms exposed to 0.4 M ethanol, comparing punctal expression levels between trained and untrained. **(B)** GLR-1::GFP punctal expression measured as Mean Total GFP expressing area/worm (±SEM) with measures normalized by distance measured along the ventral nerve cord. Measures captured between control (*n* = 15) and trained worms (0.0 M ethanol; *n* = 18) as well as control (*n* = 17) and trained (*n* = 18) worms on 0.4 M ethanol with comparisons between trained (black bars) and untrained groups (white bars). * = *p* < 0.05.

The long-term memory for habituation training protocol used here has been shown to lead to decreased punctal expression of GLR-1, an AMPA/Kainate type glutamate receptor subunit homologous to mammalian GluRs (Rose et al., 2003; Rose and Rankin, 2006). In *C. elegans*, GLR-1::GFP transgene expression in the ventral nerve cord is generally seen as bright puncta representing mostly synapses (Hart et al., 1995). To determine whether alcohol would alter this training-dependent decrease in GLR-1, punctal expression levels of GLR-1::GFP were measured 24 h after training on 0.0 M or 0.4 M ethanol (Figure 3A). Indeed, there was a significant difference in punctal expression levels between ethanol exposure groups (F_3, 64_ = 5.179, *p* < 0.01; Figure 3B). GLR-1::GFP levels for the no-ethanol group were consistent with previous findings: worms trained 24 h earlier without ethanol (0.0 M group) showed a significant decrease in total area of GLR-1::GFP punctal expression in the ventral cord compared to untrained worms (*p* < 0.05). By comparison, when exposed to 0.4 M ethanol during training, GLR-1::GFP punctal expression levels for the untrained and trained groups were not significantly different (*p* > 0.10), consistent with observations from our behavioral studies that there is a deficit in long-term memory for training in these animals. Interestingly, although GLR-1::GFP punctal expression levels from worms trained on 0.4 M ethanol did not show a training-dependent decrease in GLR-1::GFP punctal expression level, the untrained ethanol exposure group showed a significant increase in GLR-1::GFP punctal expression compared to the 0.0 M control group (*p* < 0.05).

### Alcohol-Induced Long-Term Memory Impairment Requires NLGN1

In mammals, AMPA receptor trafficking and recruitment involves a postsynaptic cell adhesion protein, neuroligin-1 (NLGN1; Heine et al., 2008; Mondin et al., 2011). NLGN1 has been shown to be involved in stabilizing AMPA receptors at synapses, and this process can occur within 2–8 h (Zeidan and Ziv, 2012). NLGN1 also binds to the post-synaptic density complex PSD-95 to interact with it and other proteins, and has been shown to play an integral role in controlling the function of excitatory synapses through AMPAR regulation (Nam and Chen, 2005). Interestingly, ethanol has also been shown to influence post-synaptic density of glutamatergic receptors (Chandler, 2003; Burnett et al., 2016). Based on these observations, we hypothesized that neuroligin might play a role in mediating the effects of ethanol on long term memory for habituation.

**Figure 4:**
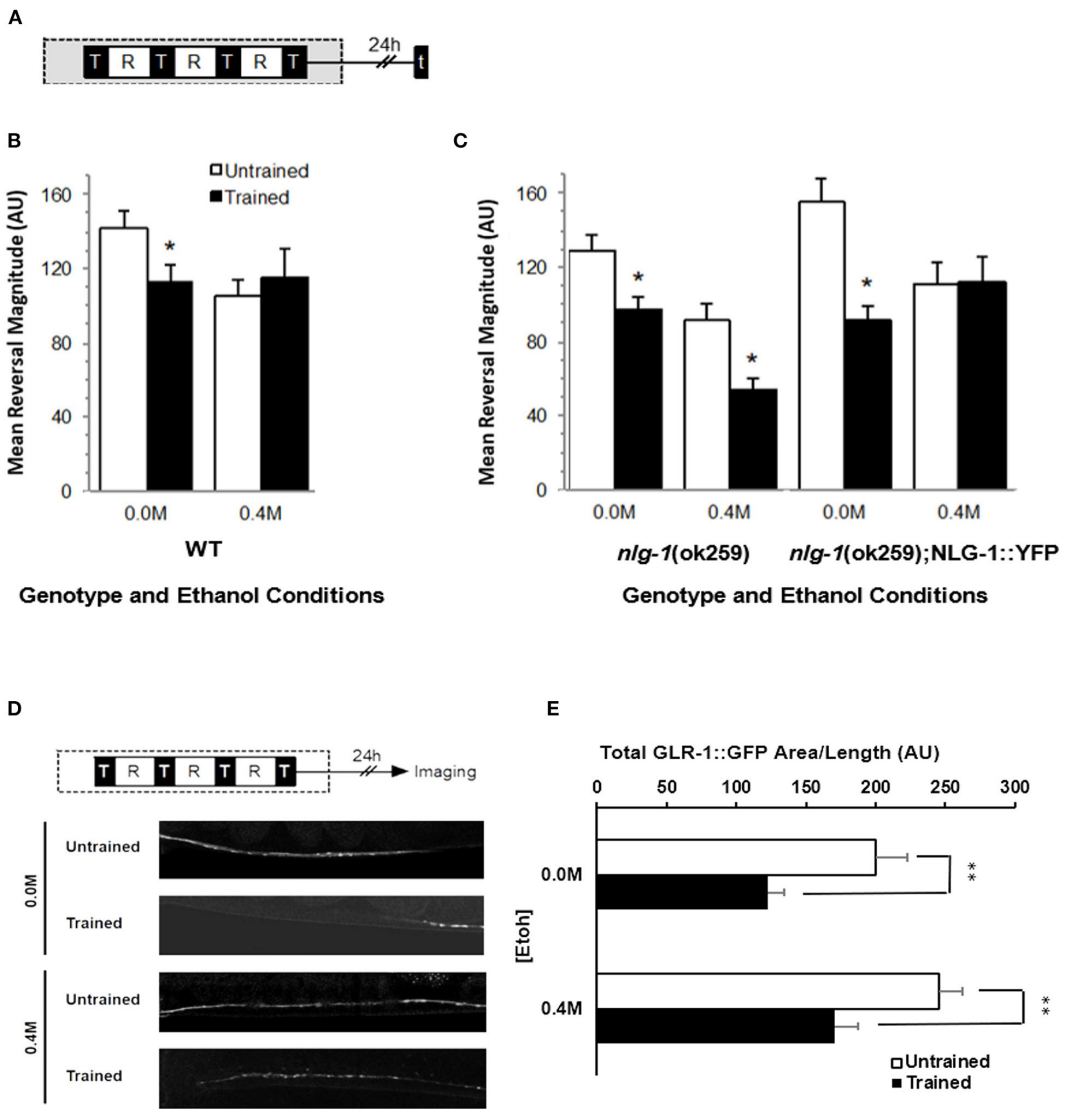
Effects of ethanol exposure during training on long-term memory in *nlg-1* mutant worms. **(A)** Long-term habituation memory training protocol with four training blocks (black squares marked by “T”) separated by 1-h rest period (white squares marked by “R”). 5 test-tap trial indicated by black square marked with “t” or GFP “imaging” was performed 24 h after training. Gray shaded area indicates period of ethanol exposure. **(B)** Mean reversal response magnitude (± SEM) to 5 test taps for trained groups (black bars) compared to untrained groups (white bars) for wild-type worms either on (0.4 M) or off (0.0 M) ethanol. **(C)** Mean reversal response magnitude (± SEM) across 5 taps of *nlg-1* mutants or *nlg-1* mutant worms expressing an NLG-1 rescue transgene. **(D)** Examples of confocal images of GLR-1::GFP punctal expression captured from *nlg-1* background worms exposed to 0.4 M ethanol, comparing expression levels between trained and untrained. **(E)** GLR-1::GFP punctal expression measured as Mean Total GFP expressing area/worm (±SEM) with measures normalized by distance measured along the ventral nerve cord. Measures captured between control (0.0 M) and 0.4 M ethanol exposure groups with comparisons between trained (black bars) and untrained groups (white bars). * = *p* < 0.05 ** = *p* < 0.01.

The *C. elegans* gene *nlg-1* encodes the worm ortholog of NLGN1 (Hunter et al., 2010). To test whether *C. elegans* neuroligin played a role in the effects of ethanol on long-term memory for habituation in *C. elegans*, we first measured reversal responses of *nlg-1*(ok259) loss of function deletion worms, 24 h after they were given long-term habituation training either on or off 0.4 M ethanol (Figure 4A). As expected, wild-type worms showed memory when trained in the absence of ethanol, but not when trained on 0.4 M ethanol (Figure 4B). Interestingly, *nlg-1*(ok259) worms that had received training had significantly smaller reversal responses to test taps compared to untrained worms when trained either on or off of 0.4 M ethanol (F_7, 529_ = 6.622, *p* < 0.01; Figure 4C) suggesting that alcohol did not disrupt memory in the absence of a functional *nlg-1* gene. This result was not due to a difference in ethanol sensitivity between wild-type and *nlg-1*(ok259) worms as locomotor speed, an indicator of drug tolerance in *C. elegans* (Davies et al., 2004), showed no difference in the presence of ethanol (Supplementary Figure 3).

To confirm that *nlg-1* expression was necessary for the ethanol-induced disruption of memory, a *nlg-1* rescue strain was also tested in which an integrated wild-type copy of *nlg-1* fused with YFP driven by the *nlg-1* endogenous promoter was expressed in the *nlg-1* mutant strain (*nlg-1*(ok259); _p_*nlg-1*::NLG-1::YFP). *nlg-1* rescue worms trained without ethanol showed significantly smaller responses to test taps indicative of memory similar to wild-type worms (*p* < 0.05), and *nlg-1* rescue worms trained on ethanol did not show long-term memory (*p* > 0.10; Figure 4C).

To support the behavioral findings, GLR-1::GFP punctal expression in *nlg-1* mutant worms was imaged 24 h after long-term habituation training and analyzed (Figure 4D). In these conditions, *nlg-1* worms showed a restored decrement in GLR-1::GFP punctal expression when trained in the presence of ethanol (*p* < 0.01), similar to GLR-1::GFP punctal expression in trained WT worms (*p* < 0.01; Figure 4E). This result indicates that the *nlg-1*(ok259) mutation seems to preserve the learning-induced decrease in GLR-1::GFP punctal expression, a decrease that appeared to be inhibited by ethanol (Figure 3B). Furthermore, there was no statistical difference in GLR-1::GFP punctal expression levels between untrained *nlg-1* or WT background worms in the presence of ethanol (*p* > 0.15). These data confirm an important role for NLG-1 in mediating the negative effects of ethanol on long-term memory for habituation and the corresponding decrease in GLR-1 punctal expression in *C. elegans*.

### Neuroligin (NLG-1) Is Necessary for the Alcohol-Induced Increase in Glutamate Receptor Expression

**Figure 5:**
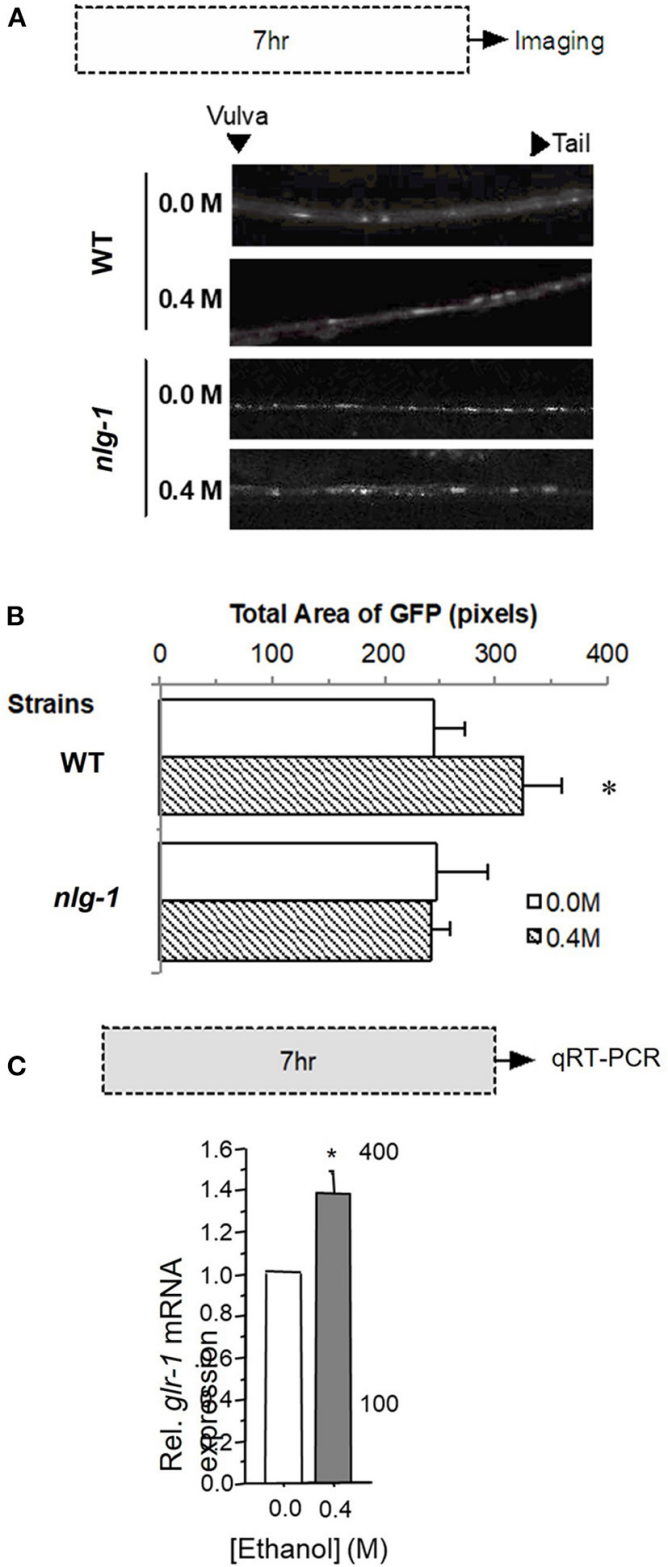
Expression of GLR-1 receptors in either a wild-type or *nlg-1* mutant worm background after 7 h ethanol exposure. **(A)** Protocol for 0.4 M ethanol exposure (gray rectangle) followed by confocal imaging and sample images captured from GLR-1::GFP worms that are either WT (for *nlg-1*) or carry a mutation in *nlg-1* exposed to either 0.0 or 0.4 M ethanol. **(B)** Mean Total GLR-1::GFP area (± SEM) for WT vs. *nlg-1* mutant worms exposed to either 0.0 or 0.4 M ethanol following 7-h exposure period. **(C)** *glr-1* mRNA levels relative to actin (*act-1*) expression (± SD) in control worms (0.0 M ethanol) compared to treatment (0.4 M ethanol) following 7-h exposure period. * = *p* < 0.05.

Because we found that in wild-type worms exposure to ethanol led to an increase in GLR-1::GFP 24 h following exposure to ethanol (Figure 3), we further investigated the characteristics of this increase by measuring GLR-1::GFP punctal expression in WT and *nlg-1* mutant worms 1 h after a 7-h exposure to 0.4 M ethanol (corresponding to the duration of ethanol exposure during long-term memory training in earlier experiments). GLR-1::GFP punctal expression in the *nlg-1* mutant worms appeared as bright puncta in the ventral nerve cord similar to that seen in wild-type worms (Figure 5A).

We found that 1 h after a 7-h 0.4 M ethanol exposure GLR-1::GFP punctal expression increased significantly compared to 0.0 M exposure in wild-type worms (F_1, 43_ = 4.941, *p* < 0.05; Figure 5B); this increase persisted for at least 24 h (Supplementary Figure 1). Interestingly, there were no significant differences in GLR-1::GFP punctal expression levels of *nlg-1* mutant worms between the 0.0 M and the 0.4 M ethanol groups (*t*_24_ = −0.022, *p* = N.S.; Figure 5B). In contrast, in *nlg-1* worms there were no significant differences between 0.0 and 0.4 M ethanol exposure groups in GLR-1::GFP punctal expression levels 7 or 24 h after ethanol exposure. These data suggest that functional neuroligin plays an important role in the ethanol-induced increase in GLR-1 expression seen in wild-type worms. The increase in GLR-1::GFP expression in wild-type worms after the 7-h ethanol exposure was confirmed with qRT-PCR which showed that glr-1 mRNA expression was 1.4 fold higher in 0.4 M ethanol treated worms than in untreated controls (*p* < 0.05; Figure 5C).

## Discussion

Our findings indicate that ethanol exposure during training interferes with long- term memory for that training. Consistent with alcohol-induced memory impairments in a number of other species including humans, we found that the effect of alcohol on memory was dose-dependent as exposure to higher concentrations of ethanol during training effectively blocked memory 24 h later (Figure 1A). Worms on a 0.4 M ethanol plate for an hour prior to training would reach the equivalent of ~0.3 percent blood alcohol concentration (BAC) estimated from internal concentration (Alaimo et al., 2012). In humans this concentration would be equivalent to consuming 6–8 drinks of alcohol, and would cause severe physical and sensory impairment. Interestingly, laboratory studies with humans on the effect of acute alcohol consumption on alcohol-induced memory blackouts by Goodwin et al. (1970) found that average peak blood alcohol concentration of individuals who showed memory disruption for events that occurred while drinking was ~0.28 percent. Therefore, the higher ethanol concentration reported here (0.4 M) should produce a sufficient level of intoxication similar to humans.

In *C. elegans* the ethanol-induced memory impairment was specific for events that occurred during ethanol exposure as ethanol given after training had no effect on memory (Figure 1B). Finally, the current study found that neither context nor internal state cues present during testing could restore memory (Figure 1C), suggesting a deficit in memory encoding. Taken together, these behavioral data further establish *C. elegans* as a valid model in which to study ethanol-induced impairments in long-term memory formation.

Although long-term memory was significantly affected by ethanol exposure during training, the effects of ethanol exposure on short-term memory for habituation were mixed. Ethanol did not affect short-term memory for habituation to stimuli presented at a short ISI (10 s) but there was mild alteration in habituation to stimuli presented at a long ISI (60 s; Figure 2B). It important to note that despite the modest change in habituation at a long ISI reported, ethanol-exposed worms still showed a significant decrement in response over time that reached an asymptotic response level that was significantly lower than naïve animals. In addition, although both 0.2 M and 0.4 M ethanol concentrations altered short-term habituation for stimuli presented at a long ISI to a similar degree, only training at 0.4 M blocked the formation of long-term memory. Thus, the effects of ethanol on long-term memory were unlikely due to a decrease in short-term habituation during training, and the mechanisms by which alcohol blocks formation of long-term memory may be distinct from those involved in impairment of short-term habituation. We earlier reported that in glr-1 mutant worms we saw normal short-term habituation and impairment of long-term memory for habituation (Rose et al., 2003).

Previous research from our lab showed that the activation of GLR-1 glutamate receptors during training is required for the behavioral expression of long-term memory for habituation that is correlated with a significant decrease in the punctal expression of GLR-1::GFP 24 h after training (Rose et al., 2003). In the current study, we found that ethanol exposure during training blocked both long-term memory for habituation as well as the decrease in GLR-1 subunit punctal expression. These results parallel previous studies that have shown experimental manipulations that block the training-induced down-regulation of GLR-1::GFP (i.e., protein synthesis inhibition between training blocks or after cue presentation) similarly block the behavioral indicators of long-term memory (Rose et al., 2003; Rose and Rankin, 2006). We hypothesized that the presence of ethanol somehow inhibited the training-induced decrease in GLR-1.

The unexpected increase in punctal GLR-1::GFP expression in the ethanol-exposed untrained condition (Figure 3B) suggested that ethanol alone increases GLR-1 expression and could potentially compete with, or counteract, any training-induced decrease in GLR-1. At a systems level, ethanol inhibits nervous system activity and as a compensatory mechanism, excitatory glutamatergic neurotransmission may be upregulated (Carpenter-Hyland et al., 2004; McCool et al., 2010; Zorumski et al., 2014). Ary et al. (2012) showed that ethanol increased GluR1 AMPA receptor subunit expression in the rodent nucleus accumbens. Work in mammalian cortical cultures found that ethanol increased expression of AMPA and NMDA receptor subunits (e.g., Hu et al., 1996; Chandler et al., 1999; Chandler, 2003), and increased GluR1 AMPA receptor subunits in the dopaminergic ventral tegmental area, specifically (Ortiz et al., 1995; Carlezon and Nestler, 2002). Thus, consistent with research in other model systems, the effects of ethanol on glutamate signaling may be the means by which ethanol affects memory.

The finding that ethanol increased the baseline level of GLR-1::GFP and impaired the training-mediated decrease in GLR-1::GFP led us to investigate the *nlg-1* gene as potentially important in mediating the effects of ethanol on memory in *C. elegans*. Neuroligins are a family of postsynaptic cell adhesion proteins that link to other synaptic proteins, including postsynaptic transmitter receptors (see Bemben et al., 2015 for review). Neuroligins are known to function in synaptogenesis (Scheiffele et al., 2000; Graf et al., 2004), synapse maturation and differentiation (Song et al., 1999; Levinson et al., 2005; Heine et al., 2008), and some forms of plasticity (Futai et al., 2007; Kim et al., 2008; Shipman and Nicoll, 2012). Because functional NLG-1 seemed to be necessary for the ethanol-induced increase in GLR-1::GFP, we tested whether *nlg-1* mutants would show a return of long-term memory and decreased GLR-1::GFP receptor expression despite ethanol exposure during training. When tested 24 h after training with ethanol, *nlg-1* mutant worms did form long-term memory for habituation (Figure 4C) and the memory-associated decrease in GLR-1::GFP punctal expression (Figure 4E). Memory was not seen in a transgenic NLG-1 rescue strain that expressed NLG-1 with its endogenous promoter. Thus, functional neuroligin plays a role in alcohol-mediated impairment of memory mechanisms.

Previous mammalian studies suggest that neuroligin is required to preserve neuronal activity-dependent changes in AMPA receptor expression levels (Zeidan and Ziv, 2012). In *C. elegans*, the single *nlg-1* gene encodes orthologs of vertebrate neuroligin isoforms (Hunter et al., 2010) and *nlg-1* mutant phenotypes in *C. elegans* can be rescued by either human or rat neuroligin orthologs (Calahorro and Ruiz-Rubio, 2012), demonstrating that worm neuroligin has high functional homology with mammalian neuroligins. When we tested the effects of ethanol on GLR-1::GFP punctal expression in *nlg-1* mutant worms we found no difference in expression levels (Figures 4E, 5B), thus confirming that functional NLG-1 protein plays an important role in the ethanol-induced increase in GLR-1::GFP. One possibility may be disruption of neuroligin-neurexin neuron-glial connections thus interfering with decreased glutamate transmission and neuron excitability by glial glutamate transporter GLT-1 (Aida et al., 2015; Katz et al., 2019; Walker et al., 2020). Alternatively, given the known roles of neuroligin (Südhof, 2008) it is possible that in *C. elegans* ethanol acts through some as yet unknown mechanism to increase *glr-1* expression levels, which perhaps require NLG-1 for stabilization. More research is needed to determine the exact mechanism by which ethanol and NLG-1 increase GLR-1::GFP expression levels.

The current study provides evidence that GLR-1 expression is increased by ethanol exposure, an effect that required the cell adhesion protein NLG-1. There is some evidence from mammalian neuron culture studies that suggest Nlgn1 mutations can reduce long-term potentiation (LTP; cellular correlate of memory; Shipman and Nicoll, 2012; Jedlicka et al., 2015); however, evidence is lacking with regards to neuroligins recruiting new AMPA receptors to synapses (a hallmark feature of LTP; Shipman and Nicoll, 2012; Bemben et al., 2015). Ethanol has been shown to either inhibit or reverse LTP (Izumi et al., 2005; Yin et al., 2007; Mishra et al., 2012; Avchalumov and Mandyam, 2020). Thus, Nlgn1 function may oppose the effects of ethanol with regards to LTP. It is difficult to test the role of NLG-1 in a glr-1 mutant in *C. elegans* as long-term memory for habituation is glutamate-dependent and completely absent in glr-1 mutant worms (Rose et al., 2003). Additional studies are needed to characterize the role of NLG-1 in ethanol-induced alterations in glutamate signaling.

Researchers that use simple model systems have identified shared sites of action for ethanol and have directly linked these findings to distinct behavioral effects. In *Drosophila*, Moore et al. (1998) identified a mutant with increased sensitivity to ethanol; this mutation disrupted the amnesiac gene, a gene originally identified in a screen for learning and memory deficits. From the current study it appears that, although habituation was modestly impacted by ethanol, the more significant deficit was seen with long-term memory. Our evidence suggests that memory mechanisms are perhaps not directly targeted by ethanol *per se*, but rather, that ethanol-induced memory impairment may result from disrupting or co-opting memory mechanisms (i.e., decreased GLR-1 expression in the case of habituation) perhaps to compensate for the depressive effects of ethanol on the nervous system.

The finding that neuroligin is involved in the effects of ethanol on AMPA receptor trafficking in several species reinforces the efficacy of how model systems can be useful to identify pathways affected by ethanol. Recent research in *Drosophila* by Petruccelli et al. (2018) demonstrated that alcohol can affect associative memories for reward through the conserved Notch molecule, another postsynaptic cell adhesion protein. In zebrafish (*Danio rerio*), Bertoncello et al. (2019) reported that ethanol acutely impaired memory consolidation for inhibitory avoidance learning; this work will hopefully lead to identification of genes critical for this effect. Taken together these data indicate that the detrimental effects of alcohol on learning and memory are highly conserved and often affect orthologous genes critical for human brain development and function.

## Data Availability Statement

The raw data supporting the conclusions of this article will be made available by the authors, without undue reservation.

## Author Contributions

JR composed multiple sections of the manuscript and revised figures. MB performed the initial long-term habituation study, confocal imaging and composed the original manuscript draft. JL and MP conducted additional confocal imaging trials and revised the manuscript. CL performed the qPCR experiments and revised the manuscript. CR conceptualized the study, consulted, supervised data collection, analysis throughout, composed, and revised all the manuscript drafts. All authors contributed to the article and approved the submitted version.

## Funding

This research was funded by an Natural Sciences and Engineering Research Council (NSERC) of Canada Discovery grant (#122216-2013,-2019) to CR. Some strains were provided by the CGC, which is funded by NIH Office of Research Infrastructure Programs (P40 OD010440).

## Conflict of Interest

The authors declare that the research was conducted in the absence of any commercial or financial relationships that could be construed as a potential conflict of interest.

## Publisher's Note

All claims expressed in this article are solely those of the authors and do not necessarily represent those of their affiliated organizations, or those of the publisher, the editors and the reviewers. Any product that may be evaluated in this article, or claim that may be made by its manufacturer, is not guaranteed or endorsed by the publisher.

## References

[B1] Aida T. Yoshida J. Nomura M. Tanimura A. Iino Y. Soma M.. (2015). Astroglial glutamate transporter deficiency increases synaptic excitability and leads to pathological repetitive behaviors in mice. Neuropsychopharmacology 40, 1569–1579. 10.1038/npp.2015.26 25662838 PMC4915262

[B2] Alaimo J. T. Davis S. J. Song S. S. Burnette C. R. Grotewiel M. Shelton K. L.. (2012). Ethanol metabolism and osmolarity modify behavioral responses to ethanol in C. elegans. Alcohol. Clin. Exp. Res. 36, 1840–1850. 10.1111/j.1530-0277.2012.01799.x 22486589 PMC3396773

[B3] Ambrose M. L. Bowden S. C. Whelan G. (2001). Working memory impairments in alcohol dependent participants without clinical amnesia. Alcohol. Clin. Exp. Res. 25, 185–191. 10.1111/j.1530-0277.2001.tb02197.x 11236831

[B4] Ary A. W. Cozzoli D. K. Finn D. A. Crabbe J. C. Dehoff M. H. Worley P. F.. (2012). Ethanol up-regulates nucleus accumbens neuronal activity dependent pentraxin (Narp): implications for alcohol-induced behavioral plasticity. Alcohol 46, 377–387. 10.1016/j.alcohol.2011.10.003 22444953 PMC3358440

[B5] Avchalumov Y. Mandyam C. D. (2020). Synaptic plasticity and its modulation by alcohol. Brain Plast. 6, 103–111. 10.3233/BPL-190089 33680849 PMC7902982

[B6] Bemben M. A. Shipman S. L. Nicoll R. A. Roche K. W. (2015). The cellular and molecular landscape of neuroligins. TINS 38, 496–505. 10.1016/j.tins.2015.06.004 26209464 PMC9381026

[B7] Bertoncello K. T. Müller T. E. Fontana B. D. Franscescon F. Gilvan Filho L. B. Rosemberg D. B. (2019). Taurine prevents memory consolidation deficits in a novel alcohol-induced blackout model in zebrafish. Prog. Neuro-Psychopharmacol. Biol. Psychiatry 93, 39–45. 10.1016/j.pnpbp.2019.03.006 30880191

[B8] Bettinger J. C. McIntire S. L. (2004). State-dependency in *C. elegans*. Genes Brain Behav. 3, 266–272. 10.1111/j.1601-183X.2004.00080.x 15344920

[B9] Burnett E. J. Chandler L. J. Trantham-Davidson H. (2016). Glutamatergic plasticity and alcohol dependence-induced alterations in reward, affect and cognition. Prog. Neuropsychopharmacol. Biol. Psychiatry 65, 309–20. 10.1016/j.pnpbp.2015.08.012 26341050 PMC4679411

[B10] Calahorro F. Ruiz-Rubio M. (2012). Functional phenotypic rescue of Caenorhabditis elegans neuroligin-deficient mutants by the human and rat NLGN1 genes. PLoS ONE 7, e39277. 10.1371/journal.pone.0039277 22723984 PMC3377638

[B11] Carlezon W. A. Jr Nestler E. (2002). Elevated levels of GluR1 in the midbrain: a trigger for sensitization to drugs of abuse? TINS 25, 610–615. 10.1016/S0166-2236(02)02289-0 12446127

[B12] Carpenter-Hyland E. P. Woodward J. J. Chandler L. J. (2004). Chronic ethanol induces synaptic but not extrasynaptic targeting of NMDA receptors. J. Neurosci. 24, 7859–7868. 10.1523/JNEUROSCI.1902-04.2004 15356198 PMC6729936

[B13] Chandler C. M. Overton J. S. Rüedi-Bettschen D. Platt D. M. (2017). “GABAA receptor subtype mechanisms and the abuse-related effects of ethanol: genetic and pharmacological evidence,” in The Neuropharmacology of Alcohol. Handbook of Experimental Pharmacology, Vol. 248, eds K. Grant and D. Lovinger (Cham: Springer). 10.1007/164_2017_80 29204713

[B14] Chandler L. J. (2003). Ethanol and brain plasticity: receptors and molecular networks of the postsynaptic density as targets of ethanol. Pharmacol. Ther. 99, 311–326. 10.1016/S0163-7258(03)00096-2 12951163

[B15] Chandler L. J. Norwood D. Sutton G. (1999). Chronic ethanol upregulates NMDA and AMPA, but not kainate receptor subunit proteins in rat primary cortical cultures. Alcohol. Clin. Exp. Res. 23, 363–370. 10.1111/j.1530-0277.1999.tb04123.x 10069569

[B16] Chastain G. (2006). Alcohol, neurotransmitter systems, and behavior. J. Gen. Psych. 133, 329–335. 10.3200/GENP.133.4.329-335 17128954

[B17] Crabbe J. C. Belknap J. K. Buck K. J. (1994). Genetic animal models of alcohol and drug abuse. Science 264, 1715–1723. 10.1126/science.8209252 8209252

[B18] Davies A. G. Bettinger J. C. Thiele T. R. Judy M. E. McIntire S. L. (2004). Natural variation in the npr-1 gene modifies ethanol responses of wild strains of C. elegans. Neuron 42, 731–743. 10.1016/j.neuron.2004.05.004 15182714

[B19] Davies A. G. Pierce-Shimomura J. T. Kim H. VanHoven M. K. Thiele T. R. Bonci A.. (2003). A central role of the BK potassium channel in behavioral responses to ethanol in C. elegans. Cell 115, 655–666. 10.1016/S0092-8674(03)00979-6 14675531

[B20] Ebrahimi C. M. Rankin C. H. (2007). Early patterned stimulation leads to changes in adult behavior and gene expression in C. elegans. Genes Brain Behav. 6, 517–528. 10.1111/j.1601-183X.2006.00278.x 17054718

[B21] Futai K. Kim M. J. Hashikawa T. Scheiffele P. Sheng M. Hayashi Y. (2007). Retrograde modulation of presynaptic release probability through signaling mediated by PSD-95–neuroligin. Nat. Neurosci. 10, 186–195. 10.1038/nn1837 17237775 PMC4755312

[B22] Gioia D. A. Alexander N. McCool B. A. (2017). Ethanol mediated inhibition of synaptic vesicle recycling at amygdala glutamate synapses is dependent upon Munc13-2. Front. Neurosci. 11, 424. 10.3389/fnins.2017.00424 28785200 PMC5519577

[B23] Gioia D. A. McCool B. (2017). Strain-dependent effects of acute alcohol on synaptic vesicle recycling and post-tetanic potentiation in medial glutamate inputs to the mouse basolateral amygdala. Alcohol. Clin. Exp. Res. 41, 735–746. 10.1111/acer.13343 28118494 PMC5378618

[B24] Goodwin D. W. Othmer E. Halikas J. A. Freemon F. (1970). Loss of short–term memory as a predictor of the alcoholic “black–out.” Nature 227, 201–202. 10.1038/227201a0 4913709

[B25] Graf E. R. Zhang X. Jin S. X. Linhoff M. W. Craig A. M. (2004). Neurexins induce differentiation of GABA and glutamate postsynaptic specializations via neuroligins. Cell 119, 1013–1026. 10.1016/j.cell.2004.11.035 15620359 PMC2826211

[B26] Grant I. (1987). Alcohol and the brain: Neuropsychological correlates. J. Consult. Clin. Psychol. 55, 310–324. 10.1037/0022-006X.55.3.310 3597943

[B27] Harris R. A. Valenzuela C. F. Brozowski S. Chuang L. Hadingham K. Whiting P. J. (1998). Adaptation of γ-aminobutyric acid type A receptors to alcohol exposure: studies with stably transfected cells. J. Pharmacol. Exp. Ther. 284, 180–188. 9435176

[B28] Hart A. C. Sims S. Kaplan J. M. (1995). Synaptic code for sensory modalities revealed by *C. elegans* GLR-1 glutamate receptor. Nature 378, 82–85. 10.1038/378082a0 7477294

[B29] Heine M. Thoumine O. Mondin M. Tessier B. Giannone G. Choquet D. (2008). Activity-independent and subunit-specific recruitment of functional AMPA receptors at neurexin/neuroligin contacts. Proc. Natl. Acad. Sci. U. S. A. 105, 20947–20952. 10.1073/pnas.0804007106 19098102 PMC2634880

[B30] Hu X. J. Follesa P. Ticku M. K. (1996). Chronic ethanol treatment produces a selective upregulation of the NMDA receptor subunit gene expression in mammalian cultured cortical neurons. Brain Res. Mol. Brain Res. 36, 211–218. 10.1016/0169-328X(95)00223-F 8965641

[B31] Hunter J. W. Mullen G. P. McManus J. R. Heatherly J. M. Duke A. Rand J. B. (2010). Neuroligin-deficient mutants of C. *elegans* have sensory processing deficits and are hypersensitive to oxidative stress and mercury toxicity. Dis. Model. Mech. 3, 366–376. 10.1242/dmm.003442 20083577 PMC4068633

[B32] Izumi Y. Nagashima K. Murayama K. Zorumski C. F. (2005). Acute effects of ethanol on hippocampal long-term potentiation and long-term depression are mediated by different mechanisms. Neuroscience 136, 509–517. 10.1016/j.neuroscience.2005.08.002 16216426

[B33] Jackson J. Donaldson D. I. Dering B. (2021). The morning after the night before: Alcohol-induced blackouts impair next day recall in sober young adults. PLoS ONE 16, e0250827. 10.1371/journal.pone.0250827 33939715 PMC8092761

[B34] Jedlicka P. Vnencak M. Krueger D. D. Jungenitz T. Brose N. Schwarzacher S. W. (2015). Neuroligin-1 regulates excitatory synaptic transmission, LTP and EPSP-spike coupling in the dentate gyrus in vivo. Brain Struct. Func. 220, 47–58 10.1007/s00429-013-0636-1 25713840

[B35] Katz M. Corson F. Keil W. Singhal A. Bae A. Lu Y.. (2019). Glutamate spillover in *C. elegans* triggers repetitive behavior through presynaptic activation of MGL-2/mGluR5. Nat. Commun. 10, 1–13. 10.1038/s41467-019-09581-4 31015396 PMC6478929

[B36] Kim J. Jung S. Y. Lee Y. K. Park S. Choi J. S. Lee C. J.. (2008). Neuroligin-1 is required for normal expression of LTP and associative fear memory in the amygdala of adult animals. Proc. Natl. Acad. Sci. 105, 9087–9092. 10.1073/pnas.0803448105 18579781 PMC2449369

[B37] Kumar S. Porcu P. Werner D. F. Matthews D. B. Diaz-Granados J. L. Helfand R. S.. (2009). The role of GABA A receptors in the acute and chronic effects of ethanol: a decade of progress. Psychopharm 205, 529–564. 10.1007/s00213-009-1562-z 19455309 PMC2814770

[B38] Lau H. L. Timbers T. A. Mahmoud R. Rankin C. H. (2013). Genetic dissection of memory for associative and non-associative learning in Caenorhabditis elegans. Genes, Brain and Behav. 12, 210–223. 23013276 10.1111/j.1601-183X.2012.00863.x

[B39] Leckliter I. N. Matarazzo J. D. (1989). The influence of age, education, IQ, gender, and alcohol abuse on Halstead-Reitan neuropsychological test battery performance. J. Clin. Psychol. 45, 484–512. 10.1002/1097-4679(198907)45:4<484::aid-jclp2270450402>3.0.co;2-l 2671047

[B40] Levinson J. N. Chéry N. Huang K. Wong T. P. Gerrow K. Kang R.. (2005). Neuroligins mediate excitatory and inhibitory synapse formation involvement of PSD-95 and neurexin-1β in neuroligin-induced synaptic specificity. J. Biol. Chem. 280, 17312–17319. 10.1074/jbc.M413812200 15723836

[B41] Liu M. Guo S. Huang D. Hu D. Wu Y. Zhou W.. (2022). Chronic alcohol exposure alters gene expression and neurodegeneration pathways in the brain of adult mice. J. Alzheimer's Dis. 86, 315–331. 10.3233/JAD-215508 35034908

[B42] McCool B. A. Christian D. T. Diaz M. R. Läck A. K. (2010). Glutamate plasticity in the drunken amygdala: the making of an anxious synapse. Int. Rev. Neurobiol. 91, 205–233. 10.1016/S0074-7742(10)91007-6 20813244 PMC3032604

[B43] Mira R. G. Lira M. Tapia-Rojas C. Rebolledo D. L. Quintanilla R. A. Cerpa W. (2020). Effect of alcohol on hippocampal-dependent plasticity and behavior: role of glutamatergic synaptic transmission. Front. Behav. Neurosci. 13, 288. 10.3389/fnbeh.2019.00288 32038190 PMC6993074

[B44] Mishra D. Zhang X. Chergui K. (2012). Ethanol disrupts the mechanisms of induction of long-term potentiation in the mouse nucleus accumbens. Alcohol. Clin. Exp. Res. 36, 2117–2125. 10.1111/j.1530-0277.2012.01824.x 22551245

[B45] Mondin M. Labrousse V. Hosy E. Heine M. Tessier B. Levet F.. (2011). Neurexin-neuroligin adhesions capture surface-diffusing AMPA receptors through PSD-95 scaffolds. J. Neurosci. 31, 13500–13515. 10.1523/JNEUROSCI.6439-10.2011 21940442 PMC6623291

[B46] Moore M. S. DeZazzo J. Luk A. Y. Tully T. Singh C. M. Heberlein U. (1998). Ethanol intoxication in *Drosophila*: genetic and pharmacological evidence for regulation by the cAMP signaling pathway. Cell 93, 997–1007. 10.1016/S0092-8674(00)81205-2 9635429

[B47] Nam C. I. Chen L. (2005). Postsynaptic assembly induced by neurexin-neuroligin interaction and neurotransmitter. PNAS 102, 6137–6142. 10.1073/pnas.0502038102 15837930 PMC1087954

[B48] Ortiz J. Fitzgerald L. W. Charlton M. Lane S. Trevisan L. Guitart X.. (1995). Biochemical actions of chronic ethanol exposure in the mesolimbic dopamine system. Synapse 21, 289–298. 10.1002/syn.890210403 8869159

[B49] Petruccelli E. Feyder M. Ledru N. Jaques Y. Anderson E. Kaun K. R. (2018). Alcohol activates scabrous-notch to influence associated memories. Neuron 100, 1209–1223. 10.1016/j.neuron.2018.10.005 30482693 PMC6323638

[B50] Rankin C. H. (2000). Context conditioning in habituation in the nematode Caenorhabditis elegans. Behav. Neurosci. 114, 496–505. 10.1037/0735-7044.114.3.496 10883800

[B51] Rao P. S. S. Bell R. L. Engleman E. A. Sari Y. (2015). Targeting glutamate uptake to treat alcohol use disorders. Front. Neurosci. 9, 144. 10.3389/fnins.2015.00144 25954150 PMC4407613

[B52] Robinson B. G. Khurana S. Kuperman A. Atkinson N. S. (2012). Neural adaptation leads to cognitive ethanol dependence. Curr. Biol. 22, 2338–2341. 10.1016/j.cub.2012.10.038 23200990 PMC3528820

[B53] Rose J. K. Kaun K. R. Chen S. H. Rankin C. H. (2003). GLR-1, a non-NMDA glutamate receptor homolog, is critical for long-term memory in Caenorhabditis elegans. J. Neurosci. 23, 9595–9599. 10.1523/JNEUROSCI.23-29-09595.2003 14573539 PMC6740458

[B54] Rose J. K. Kaun K. R. Rankin C. H. (2002). A new group-training procedure for habituation demonstrates that presynaptic glutamate release contributes to long-term memory in Caenorhabditis elegans. Learn. Mem. 9, 130–137. 10.1101/lm.46802 12075001 PMC182588

[B55] Rose J. K. Rankin C. H. (2001). Analyses of habituation in Caenorhabditis elegans. Learn. Mem. 8, 63–69. 10.1101/lm.37801 11274251

[B56] Rose J. K. Rankin C. H. (2006). Blocking memory reconsolidation reverses memory-associated changes in glutamate receptor expression. J. Neurosci. 26, 11582–11587. 10.1523/JNEUROSCI.2049-06.2006 17093079 PMC6674789

[B57] Salling M. C. Faccidomo S. P. Li C. Psilos K. Galunas C. Spanos M.. (2016). Moderate alcohol drinking and the amygdala proteome: identification and validation of calcium/calmodulin dependent kinase II and AMPA receptor activity as novel molecular mechanisms of the positive reinforcing effects of alcohol. Biol. Psychiatry 79, 430–442. 10.1016/j.biopsych.2014.10.020 25579851 PMC4417085

[B58] Scheiffele P. Fan J. Choih J. Fetter R. Serafini T. (2000). Neuroligin expressed in nonneuronal cells triggers presynaptic development in contacting axons. Cell 101, 657–669. 10.1016/S0092-8674(00)80877-6 10892652

[B59] Selby M. J. Azrin R. L. (1998). Neuropsychological functioning in drug abusers. Drug Alcohol. Depend. 50, 39–45. 10.1016/S0376-8716(98)00002-7 9589271

[B60] Shipman S. L. Nicoll R. A. (2012). A subtype-specific function for the extracellular domain of neuroligin 1 in hippocampal LTP. Neuron 76, 309–316 10.1016/j.neuron.2012.07.024 23083734 PMC3998838

[B61] Song J. Y. Ichtchenko K. Südhof T. C. Brose N. (1999). Neuroligin 1 is a postsynaptic cell-adhesion molecule of excitatory synapses. Proc. Natl. Acad. Sci. 96, 1100–1105. 10.1073/pnas.96.3.1100 9927700 PMC15357

[B62] Südhof T. C. (2008). Neuroligins and neurexins link synaptic function to cognitive disease. Nature 455, 903–911. 10.1038/nature07456 18923512 PMC2673233

[B63] Valenzuela C. F. (1997). Alcohol and neurotransmitter interactions. Alcohol. Health Res. World 21, 144–148. 15704351 PMC6826822

[B64] Walker C. D. Risher W. C. Risher M. L. (2020). Regulation of synaptic development by astrocyte signaling factors and their emerging roles in substance abuse. Cells 9, 297. 10.3390/cells9020297 31991879 PMC7072591

[B65] White A. M. Matthews D. B. Best P. J. (2000). Ethanol, memory, and hippocampal function: a review of recent findings. Hippocampus 10, 88–93. 10706220 10.1002/(SICI)1098-1063(2000)10:1<88::AID-HIPO10>3.0.CO;2-L

[B66] Woodward Hopf F. Mangieri R. A. (2018). “Do alcohol-related AMPA-type glutamate receptor adaptations promote intake?” in The Neuropharmacology of Alcohol, (Springer, Cham), 157–186. 10.1007/164_2018_105 29675583

[B67] Yin H. H. Park B. S. Adermark L. Lovinger D. M. (2007). Ethanol reverses the direction of long-term synaptic plasticity in the dorsomedial striatum. Eur. J. Neurosci. 25, 3226–3232. 10.1111/j.1460-9568.2007.05606.x 17552991

[B68] Zeidan A. Ziv N. E. (2012). Neuroligin-1 loss is associated with reduced tenacity of excitatory synapses. PloS ONE 7, e42314 10.1371/journal.pone.0042314 22860111 PMC3409177

[B69] Zorumski C. F. Mennerick S. Izumi Y. (2014). Acute and chronic effects of ethanol on learning-related synaptic plasticity. Alcohol 48, 1–17. 10.1016/j.alcohol.2013.09.045 24447472 PMC3923188

