# Supplementary material for: Neuroligin Plays a Role in Ethanol-Induced Disruption of Memory and Corresponding Modulation of Glutamate Receptor Expression
Source: Front Behav Neurosci. 2022 May 26;16:908630. doi: 10.3389/fnbeh.2022.908630 (PMC9204643; doi:10.3389/fnbeh.2022.908630)
Supplement: Supplementary file 1 [file Data_Sheet_1.docx]

Supplemental Fig. 1


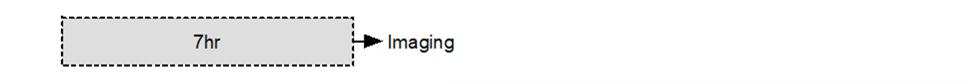

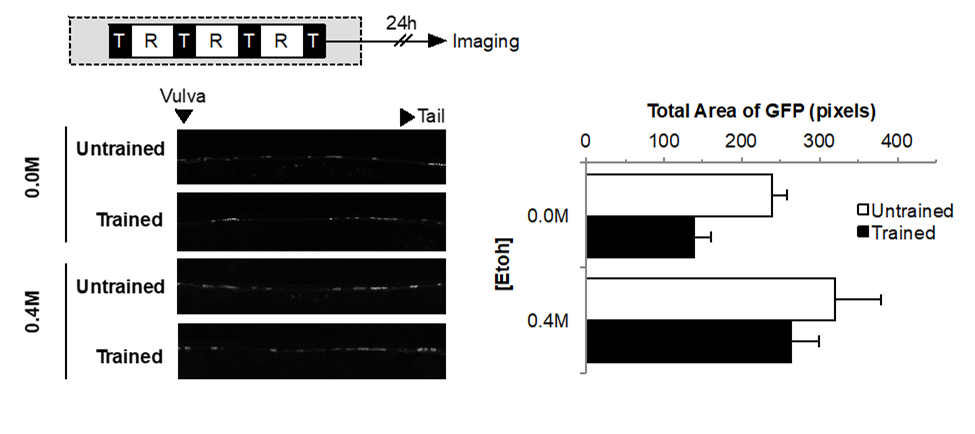

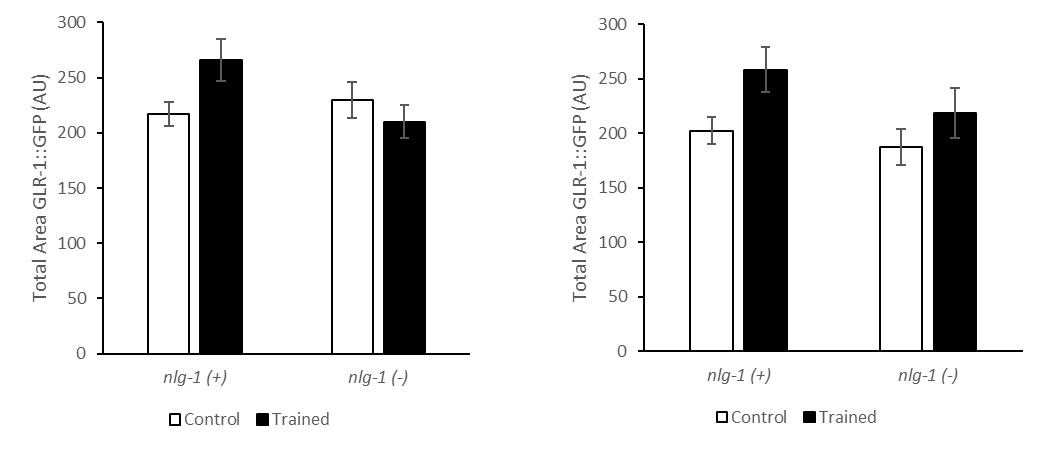


*

WT *nlg-1*

0.4 M ethanol

**Supplemental Fig. 1** *glr-1* expression measured after ethanol exposure. Protocol for 0.4 M ethanol exposure (grey rectangle) with confocal imaging occurring at 24 hours after ethanol exposure. GLR-1::GFP expression measured as Mean Total GFP expressing area/worm (±SEM) with measures normalized by distance along the ventral nerve cord. Measures captured between control group and 0.4 M ethanol exposure group for wild-type versus *nlg-1* mutant worms with comparisons between trained (black bars) and untrained (white bars). * = p < 0.05

Supplemental Fig. 2


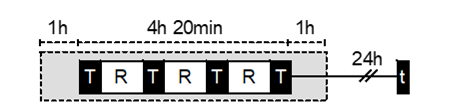

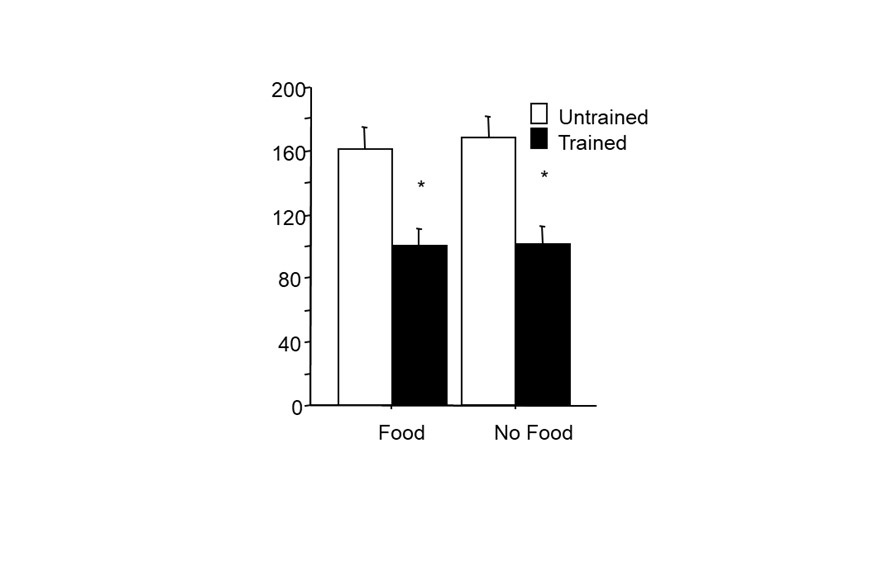


**Mean Reversal Magnitude (AU)**

**Food Condition During Training**

**Supplemental Fig. 2** Food conditions during training have no effect on long-term memory for habituation. Top shows illustration of corresponding training and ethanol exposure protocol where grey boxes indicate time of ethanol exposure while black boxes indicate training periods denoted by “T” and test periods denoted by “t”. White squares indicate rest periods denoted by “R”. Mean reversal response magnitude (±SEM) for worms that received training with ‘Food’ versus ‘no Food’ comparing between untrained (white bars) to trained worms (black bars). * = *p* < 0.05

Supplemental Fig. 3


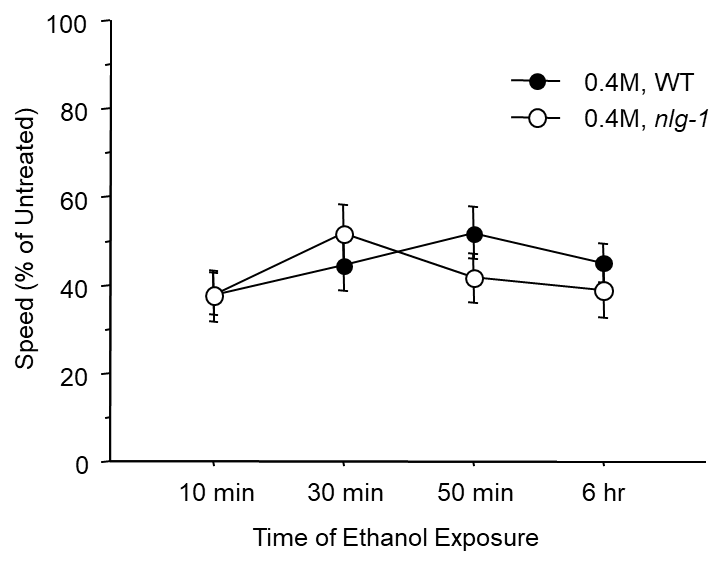


**Supplemental Fig. 3** Ethanol sensitivity measured as locomotion speed in wild-type and *nlg-1* mutant worms. Mean locomotion speed (± SEM) of four-day-old wild-type and *nlg-1* mutant worms during 0.4 M ethanol exposure observed at 10 minutes, 30 minutes, 50 minutes, and 6 hours (white circles) relative to untreated worms (black circles). The distance each worm traveled was measured in 10 s interval bins at each time point. To control for differences in basal speed across different strains, relative speed was calculated for each strain (treated average speed/untreated average speed x 100).
